# HO-1 induced autophagy protects against IL-1 β-mediated apoptosis in human nucleus pulposus cells by inhibiting NF-κB

**DOI:** 10.18632/aging.102753

**Published:** 2020-02-04

**Authors:** Luetao Zou, Hongyan Lei, Jieliang Shen, Xulin Liu, Xiang Zhang, Longxi Wu, Jie Hao, Wei Jiang, Zhenming Hu

**Affiliations:** 1 Department of Orthopedics, The First Affiliated Hospital of Chongqing Medical University, Chongqing 400016, China; 2 Department of the First Clinical Medicine, Chongqing Medical University, Chongqing 400016, China

**Keywords:** Heme oxygenase-1 (HO-1), nucleus pulposus cells (NPCs), autophagy, apoptosis, Beclin-1/PI3KC3 complex

## Abstract

In this study, we investigated the role of heme oxygenase-1 (HO-1) in intervertebral disc degeneration (IDD) by assessing the effects of HO-1 overexpression on IL-1β-induced apoptosis in nucleus pulposus cells (NPCs). Immunohistochemical staining showed HO-1 expression to be lower in NPCs from IDD patients than from patients with lumbar vertebral fractures (LVF). Western blot analysis showed HO-1 and LC3-II/I levels to be lower in NP tissues from IDD patients than from LVF patients, suggesting suppression of autophagy in degenerative intervertebral disc. Consistent with that idea, autophagy was increased in HO-1-overexpressing NPCs while IL-1β-induced apoptosis was reduced. These effects were reversed by treatment with the early autophagy inhibitor 3-methyl adenine, which suggests HO-1-induced autophagy suppresses IL-1β-induced apoptosis in NPCs. HO-1 overexpression promoted autophagy by increasing levels of Beclin-1/PI3KC3 complex. Phospho-P65 levels were lower in HO-1-overexpressing NPCs, suggesting inhibition of NF-κB-mediated apoptosis. Our study thus demonstrates that HO-1 promotes autophagy by enhancing formation of Beclin-1/PI3KC3 complex and suppresses IL-1β-induced apoptosis by inhibiting NF-κB. We suggest that HO-1 is a potential therapeutic target to alleviate IDD.

## INTRODUCTION

Intervertebral disc degeneration (IDD) is a major reason for low back pain (LBP) that affects most people at some point in their lifetime [1–3]. The main causative factors for IDD include nutritional deficits, excessive load, aging, and inflammation [4]. High levels of pro-inflammatory cytokines such as IL-1β activate signaling pathways that induce apoptosis of the nucleus pulposus cells (NPCs) in the degenerative intervertebral disc [5, 6].

Autophagy is a highly conserved process through which eukaryotic cells recycle cellular components, including organelles and proteins [7]. Macroautophagy is the best studied form of autophagy, which involves formation of autophagosomes that capture and degrade long lived, damaged, aggregated, and misfolded proteins or organelles [8, 9]. The role of autophagy in IDD is controversial. Studies have reported increased as well as decreased levels of autophagy in the cellular components of the degenerative intervertebral disc [10, 11]. SIRT1 promotes autophagy in degenerative NPC’s and protects against apoptosis [12]. Conversely, TGF-β1 protects against apoptosis in serum-starved annulus fibrosus cells by downregulating excessive autophagy [13].

Heme oxygenase-1 (HO-1) is a stress-inducible enzyme that catalyzes the first and rate-limiting step of heme degradation [14, 15]. HO-1 is associated with antioxidant, anti-apoptotic and anti-inflammatory functions, and is involved in maintaining cellular redox [16]. Several *in vitro* studies have shown that HO-1 is upregulated by inflammatory mediators such as IL-1, TNF-α, LPS, and ROS [17, 18]. Our previous study demonstrated that HO-1 suppresses IL-1β-induced apoptosis in human degenerative NPCs through the NF-κB pathway [19]. Previous studies show that HO-1-mediated autophagy protects against cell death in hepatocytes [20] and pulmonary endothelial cells [21]. However, the regulation of autophagy by HO-1 in NPCs has not been reported.

Phosphoinositide 3-kinases (PI3Ks) are an integral part of intracellular signal transduction pathways that regulate several biological functions including autophagy [22]. The class III PI3-kinase (PI3KC3) is critical for autophagy initiation [23]. HO-1 induces autophagy in the hepatocytes and kidney proximal tubular cells by activating PI3KC3 [20, 24]. Autophagy initiation involves formation of the Beclin-1/PI3KC3 complex [25, 26]. We previously showed that autophagy suppresses apoptosis in human degenerative NPCs [12].

HO-1 suppresses apoptosis by inhibiting the NF-κB pathway in cardiac ischemia and reperfusion and rheumatoid arthritis synovial fibroblasts [27, 28]. Zhongyi et al demonstrated that NF-κB signaling pathway was a key mediator of IDD [29]. Furthermore, PI3K regulates inflammatory responses and inhibits apoptosis in the NPCs [30, 31]. However, the link between HO-1, Beclin-1/PI3KC3 complex mediated autophagy, NF-κB signaling pathway, and apoptosis of NPCs is not established.

Therefore, in this study, we investigated the mechanism by which HO-1 regulates apoptosis in degenerative human NPCs.

## RESULTS

### NP tissues from IDD patients show reduced expression of HO-1 and autophagy compared with those from LVF patients

**Figure 1:**
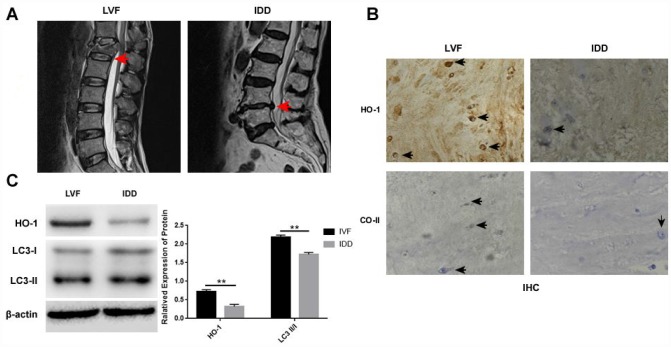
**Distinct morphology and HO-1 expression in NP tissues isolated from LVF and IDD groups.** (**A**) Representative lumbar MRI photographs show the grade I and grade V intervertebral discs in the LVF (left) and IDD (right) patient tissues, respectively, as indicted by the red arrows. The tissues were graded using the Pfirrmann’s grading system. (**B**) Immunohistochemical staining shows the expression of HO-1 and collagen II expression in the NPCs from LVF and IDD patients. The black arrows indicate positively staining cells. (**C**) Western blot shows the proteins expressions of HO-1 and LC3-II/I in NP tissue samples from LVF and IDD patients. Note: The data represents mean ± SD of three experiments; **p<0.01.

Immunohistochemical (IHC) analysis of NP tissues showed that HO-1 and collagen II positive cells were significantly reduced in the IDD group compared the LVF group (Figure 1A–1B). Moreover, western blot analysis showed that HO-1 and LC3-II/I protein levels were significantly lower in the IDD group than in the LVF group (Figure 1C).

### IL-1β induces apoptosis of NPCs in the presence of 1% FBS

**Figure 2:**
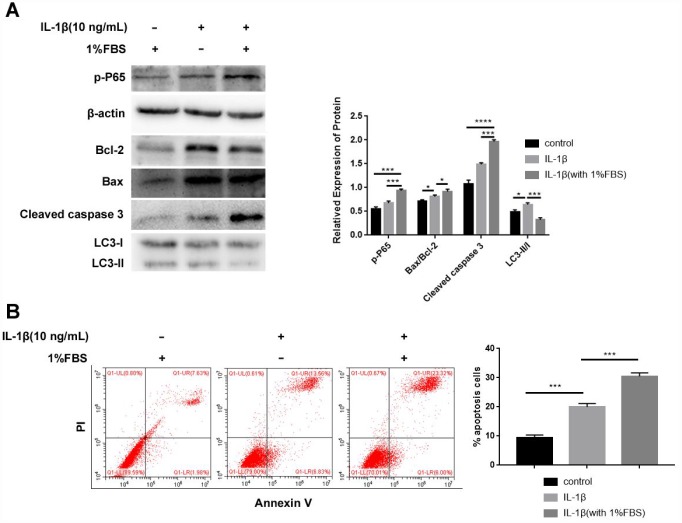
**IL-1β treatment inhibits autophagy and enhances apoptosis in the human NPCs.** (**A**) Western blot shows the proteins expressions of p-P65, Bax/Bcl-2, Cleaved caspase 3 and LC3-II/I in NPCs after IL-1β (10ng/mL) treatment with or without 1% FBS. Note: The data represent mean ± SD of three experiments; ****p<0.0001, ***p<0.001 and *p<0.05. (**B**) Flow cytometry shows the percentage of apoptotic cells in NPCs after IL-1β treatment with or without 1% FBS. Note: The data represents mean ± SD of three experiments; ***p<0.001.

A previous study showed that IL-1β induces cellular apoptosis in NPCs under 0% fetal bovine serum (FBS) but not 10% FBS condition [32]. Our preliminary experimental results showed that NPCs apoptosis increased after IL-1β treatment under 0% FBS but most of cells went from adherent to floating when recombinant adenoviral vector construct containing HO-1 (Ad-HO-1) transfected. When 1% FBS added, IL-1β can still induce apoptosis effectively, furthermore, Ad-HO-1 transfection and follow up experiments can proceed smoothly. Western blot analysis showed higher phospho-P65, Bax/Bcl-2 and Cleaved caspase3 expression, but reduced LC3-II/I levels in NPCs treated with IL-1β plus 1% FBS compared to NPCs treated with IL-1β alone (Figure 2A). Moreover, flow cytometry analysis showed that the apoptotic rate was significantly higher in the NPCs treated with IL-1β plus 1% FBS compared to NPCs treated with IL-1β alone (Figure 2B). These data showed that IL-1β induced apoptosis when NPCs were cultured in medium containing 1% FBS.

### HO-1 overexpression promotes autophagy and decreases IL-1β induced apoptosis in human NPCs

Previous reports have shown that HO-1 inhibits apoptosis by inducing autophagy [33]. Therefore, we overexpressed HO-1 in NPCs using recombinant adenoviral vector construct containing HO-1 (Ad-HO-1), and analyzed the status of autophagy and apoptosis in NPCs treated with IL-1β and 1% FBS.

**Figure 3:**
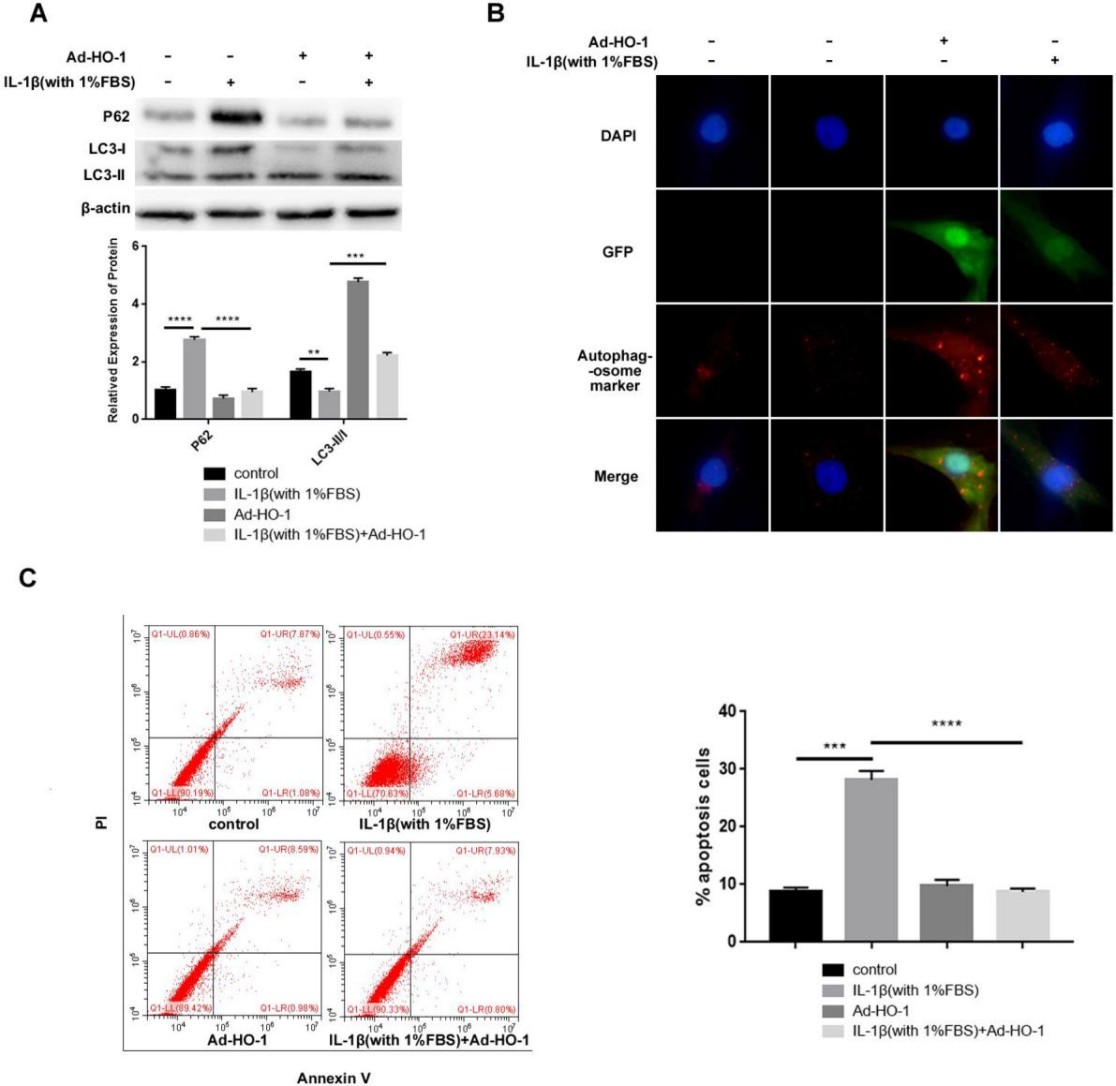
**HO-1 overexpression suppresses apoptosis induced by IL-1β in the human NPCs.** (**A**) Western blot shows the expressions of P62 and LC3-II/I protein levels in NPCs, which were transfected with Ad-HO-1 for 48 h and then stimulated with IL-1β (10ng/mL) plus 1% FBS for 24 h. Note: The data represents mean ± SD of three experiments; ****p<0.0001, ***p<0.001 and **p<0.01. (**B**) Immunofluorescence assay shows formation of autophagosomes in HO-1 overexpressing NPCs stimulated with IL-1β (10ng/mL) plus 1% FBS for 24 h. (**C**) Flow cytometry shows percentage apoptosis of HO-1 overexpressing NPCs after treatment with IL-1β (10ng/mL) plus 1% FBS for 24 h. Note: The data represents mean ± SD of three experiments; ****p<0.0001 and ***p<0.001.

HO-1 overexpressing NPCs treated with IL-1β and 1% FBS showed increased LC3-II/I and decreased P62 levels than NPCs treated with IL-1β and 1% FBS (Figure 3A). Immunofluorescent assays showed significantly higher number of autophagosomes in the HO-1 overexpressing NPCs compared with the controls when treated with IL-1β and 1% FBS (Figure 3B). Flow cytometry analysis showed significantly reduced apoptosis in the HO-1 overexpressing NPCs compared with the controls when treated with IL-1β and 1% FBS (Figure 3C). Overall, these data suggest that HO-1 overexpression induces autophagy and suppresses autophagy in the human NPCs.

### HO-1 upregulation promotes autophagy in human NPCs

**Figure 4:**
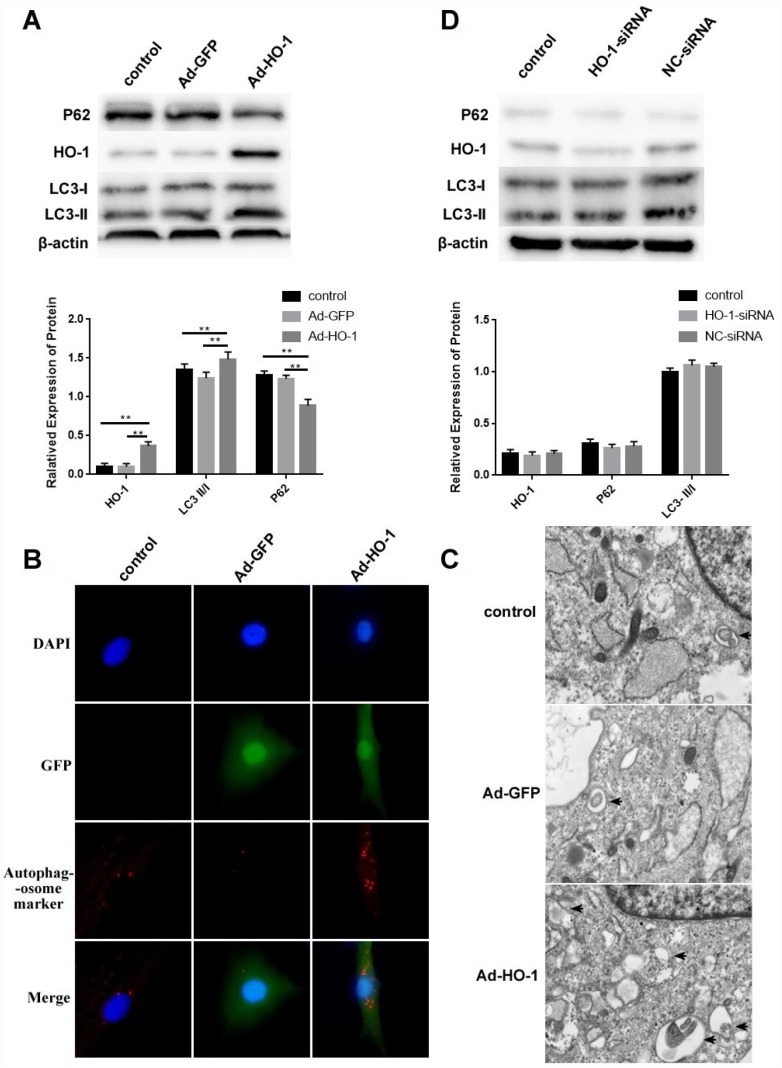
**HO-1 overexpression induces autophagy in human NPCs.** (**A**) Western bolt shows HO-1, P62 and LC3-II/I protein levels in control and HO-1 overexpressing NPCs. HO-1 overexpressing NPCs were generated by transfecting Ad-HO-1 for 48 h. Note: The data represent mean ± SD of three experiments; **p<0.01. (**B**) Immunofluorescence assay results show autophagosome formation based on staining of the control and HO-1 overexpressing NPCs with antibodies against the autophagosome marker protein SQSTM1/P62. (**C**) Transmission electron micrographs show characteristic double-membrane autophagosome formation (black arrows) in control and HO-1 overexpressing NPCs. (**D**) Western bolt shows HO-1, P62 and LC3-II/I protein levels in control and HO-1 siRNA transfected NPCs. As shown, there is no significant difference in the levels of these proteins in all experimental groups. Note: The data represent mean ± SD of three experiments.

Next, we compared the effects of HO-1 overexpression and knockdown in NPCs using Ad-HO-1 and small interference RNA against HO-1 (HO-1-siRNA). Western blot analysis showed increased expression of HO-1 and LC3-II/I, and decreased P62 levels in HO-1 overexpressing NPCs (with Ad-HO-1) compared to the controls (Figure 4A). Immunofluorescence assays and Transmission electron microscopy (TEM) analysis demonstrated that autophagosome formation was significantly increased in the Ad-HO-1 NPCs compared with the controls (Figure 4B–4C). HO-1-knockdown NPCs did not show any significant changes in the expression of HO-1, LC3-II/I and P62 compared with the controls (Figure 4D).

### Blocking autophagy inhibits the anti-apoptotic effect of HO-1, resulting in an increased apoptosis in human NPCs

**Figure 5:**
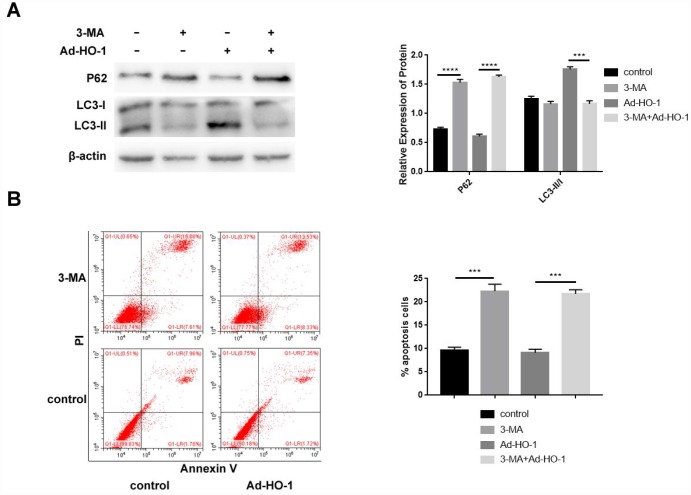
**Autophagy inhibition by 3-MA enhances apoptosis in HO-1 overexpressing NPCs.** (**A**) Western blot shows P62 and LC3-II/I protein levels in HO-1 overexpressing NPCs treated with or without 10 mM 3-MA. Briefly, NPCs were transfected with Ad-HO-1 for 48 h and then treated with 10 mM 3-MA to inhibit autophagy for 24 h. (**B**) Flow cytometry shows apoptotic rate of cells when HO-1 overexpressing NPCs were treated with or without 10 mM 3-MA. Note: All the experiments were repeated at least three times independently; ****P<0.0001 and ***p<0.001.

We analyzed if HO-1 inhibits apoptosis by enhancing autophagy by treating control and HO-1 overexpressing NPCs with the autophagy inhibitor, 3-Methyladenine (3-MA). Western blot results showed significantly decreased LC3-II/I and increased P62 levels in the compared with the untreated controls, but LC3-II/I and P62 levels did not change in the 3-MA treated HO-1 overexpressing NPCs compared with the 3-MA treated alone (Figure 5A).

Flow cytometry analysis showed that significantly increased rate of apoptosis in the 3-MA treated NPCs compared with the untreated controls, but the apoptotic rate did not significantly change in the 3-MA treated HO-1 overexpressing NPCs compared with the 3-MA treated alone (Figure 5B). These data suggest that HO-1 decreases apoptosis by inducing autophagy in human NPCs.

### Upregulation of HO-1 increases the formation of the Beclin-1/PI3KC3 complex

**Figure 6:**
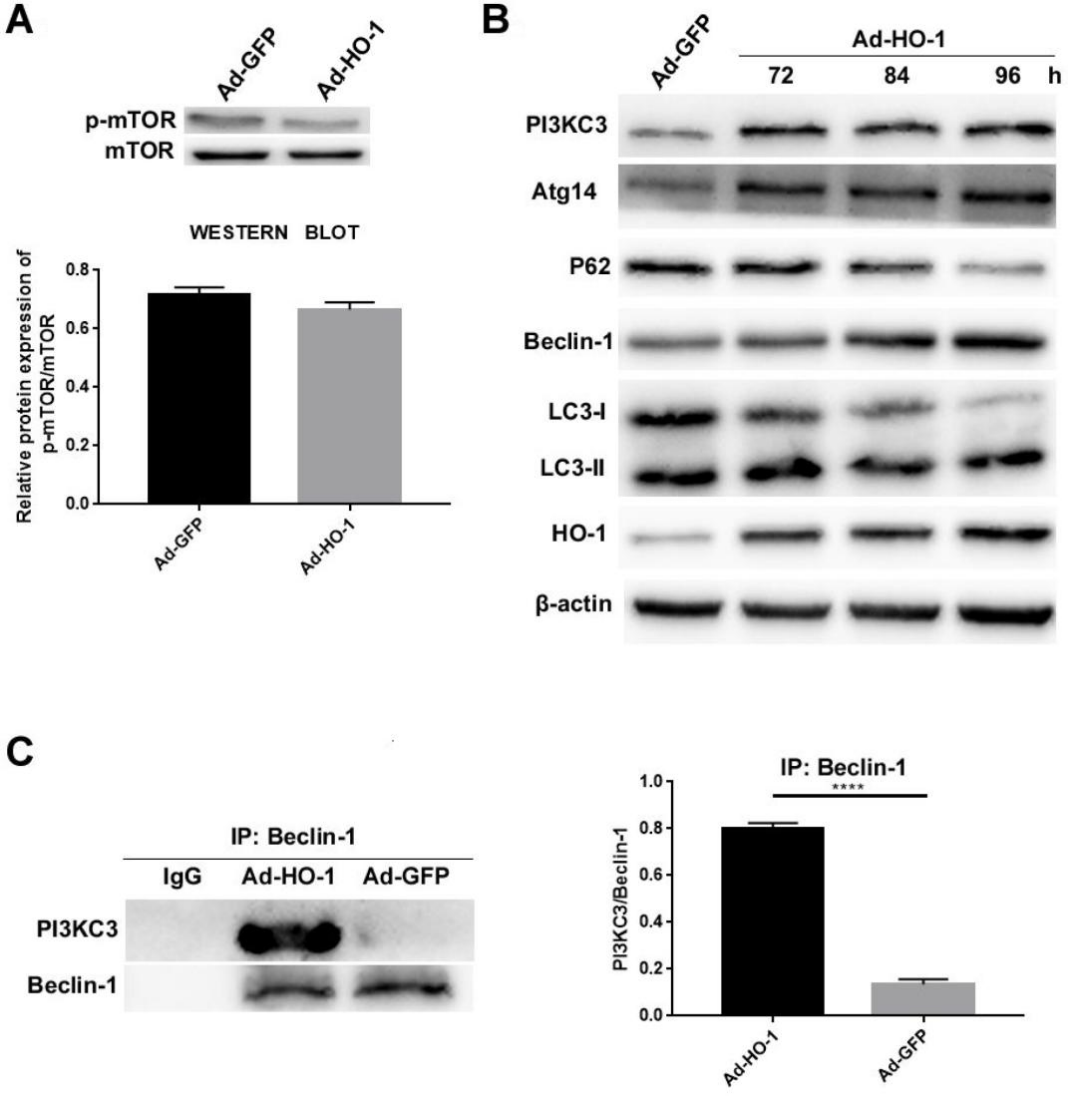
**HO-1 overexpressing NPCs show elevated levels of the Beclin-1/PI3KC3 complex.** (**A**) Western blot shows the protein levels of phospho-mTOR in NPCs transfected with Ad-HO-1 and Ad-GFP. As shown, phospho-mTOR levels are comparatively similar in both Ad-HO-1 and Ad-GFP groups. (**B**) Western blot analysis of HO-1, PI3KC3, Atg14, Beclin-1, P62 and LC3-II/I protein levels in NPCs cultured for 72 h, 84 h or 96 h after Ad-HO-1 transfection. (**C**) Immunoprecipitation assay results show the amount of Beclin-1/PI3KC3 complex in HO-1 overexpressing NPCs compared with controls. Briefly, the cell lysates were immunoprecipitated (IP) with the anti-Beclin-1 antibody and the immunoprecipitated proteins were analyzed by western blotting using the anti-PI3KC3 antibody. Note: Data are represented as mean ± SD of three independent experiments; ****p<0.0001.

Next, we analyzed the autophagy pathways activated by HO-1. First, we examined the effects of HO-1 overexpression on the status of mTOR activation in NPCs. The mTOR kinase is a well-known regulator of autophagy in eukaryotic cells [34]. We observed that the levels of mTOR and phospho-mTOR were similar in the control and HO-1 overexpressing NPCs (Figure 6A). This suggests that mTOR is not involved in the regulation of autophagy by HO-1.

Next, we analyzed the status of the Beclin-1/PI3KC3 complex, a key regulator of autophagy. PI3KC3 protein levels were significantly upregulated in the HO-1 overexpressing NPCs compared to the controls (Figure 6B). Moreover, HO-1 overexpressing NPCs showed decreased expression of P62 and increased Beclin-1, LC3-II/I and Atg14 expression compared with the controls (Figure 6B). Furthermore, the levels of PI3KC3, Beclin-1, LC3-II/I and ATG14 were increased and P62 was decreased in HO-1 overexpressing cells analyzed at 72 h, 84 h, and 96 h after Ad-HO-1 transfection (Figure 6B). Immunoprecipitation assays showed that Beclin-1/PI3KC3 complex levels were significantly increased in the HO-1 overexpressing NPCs compared with the controls (Figure 6C).

### HO-1 inhibits NF-κB signaling pathway in the human NPCs

**Figure 7:**
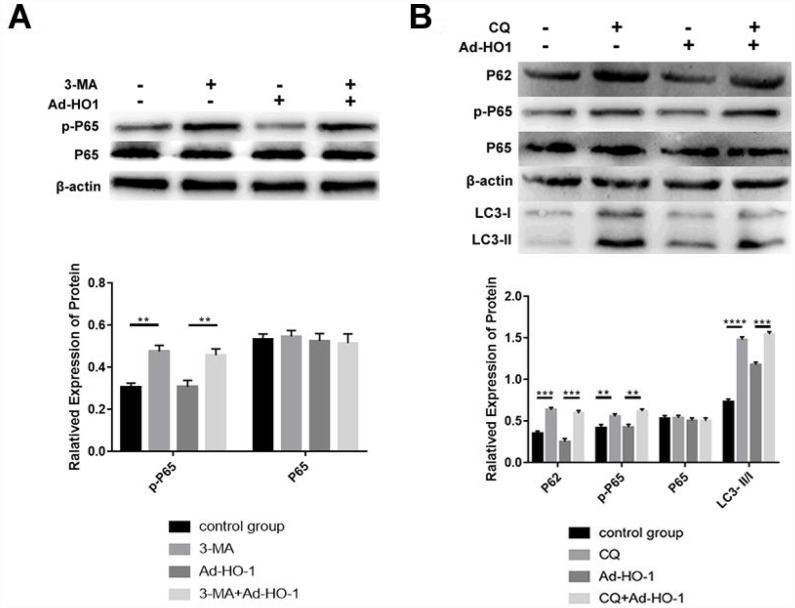
**Autophagy induced by HO-1 inhibits the NF-κB pathway in NPCs.** (**A**) Western blot analysis shows P65 and phospho-P65 protein alevels in NPCs transfected with Ad-HO-1 for 48 h and then treated with or without 10mM 3-MA for 24 h to inhibit autophagy. (**B**) Western blot analysis shows P65, p-P65, P62 and LC3-II/I protein levels in NPCs treated with 10mM CQ for 24 h after transfection with Ad-HO-1 for 48 h. Note: The data represent mean ± SD of three independent experiments; ****P<0.0001 and ***p<0.001.

We analyzed the role of the NF-κB pathway in HO-1-mediated autophagy by treating HO-1 overexpressing NPCs with the autophagy inhibitor with 3-MA and chloroquine (terminal autophagy inhibitor, CQ) to inhibit autophagy at different stages after Ad-HO-1 transfection. Western blot analysis showed that the levels of phospho-P65 (p-P65) decreased significantly in the HO-1 overexpressing NPCs compared with the untreated NPCs (Figure 7A). Moreover, 3-MA-treatment increased phospho-P65 levels in both HO-1 overexpressing and control NPCs compared with the untreated controls (Figure 7A). This suggests that the NF-κB pathway was downstream of the Beclin-1/PI3KC3 complex.

Western Blot analysis showed that treatment with CQ significantly increased the levels of P62, phospho-P65, and LC3-II/I in both HO-1 overexpressing and control NPCs compared with untreated controls (Figure 7B). This suggests that activation of autophagy by HO-1 inhibits NF-κB in human NPCs (Figure 7B).

**Figure 8:**
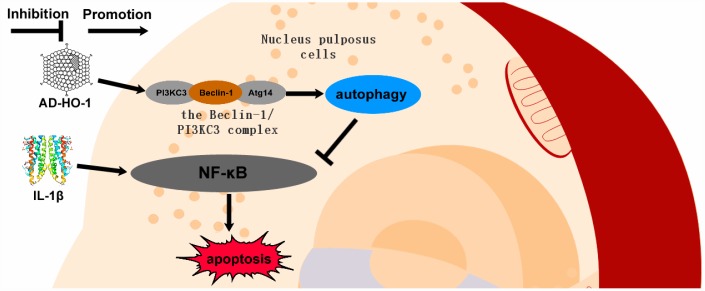
**Schematic diagram shows potential mechanism of action of HO-1.** HO-1 promotes autophagy by increasing the formation of the Beclin-1/PI3KC3 complex. HO-1 induced autophagy protects against apoptosis of human NPCs by inhibiting NF-κB.

Taken together, our data suggests that HO-1 promotes autophagy in human NPCs by increasing the formation of the Beclin-1/PI3KC3 complex and suppresses apoptosis by inhibiting the NF-κB pathway (Figure 8).

## DISCUSSION

Apoptosis of NPCs results in intervertebral disc degeneration, a common cause of low back pain [6]. Current evidence suggests that autophagy is a key regulator of apoptosis [9, 11]. HO-1 is a key factor that promotes autophagy in several cell types [20, 21]. We previously showed that HO-1 inhibits IL-1β-induced apoptosis in the human degenerative NPCs via the NF-κB pathway [19]. In this study, we demonstrate that upregulation of HO-1 suppresses IL-1β-induced apoptosis of NPCs via NF-κB by activating autophagy through the Beclin-1/PI3KC3 complex.

Pro-inflammatory cytokines, such as IL-1β and TNF-α, enhance extracellular matrix (ECM) degradation during IDD by inducing the production of matrix metallopeptidases (MMPs) and a disintegrin and metalloproteinase with thrombospondin motifs (ADAMTS) [33]. Our results show that the NPCs isolated from the IDD patients show decreased LC3-II/I expression compared with the NPCs isolated from the LVF group; moreover, collagen II levels in the extracellular matrix are lower in the IDD group compared with the LVF group. Previous studies suggest IL-1β induces cellular apoptosis in NPCs under 0% fetal bovine serum (FBS) but not 10% FBS conditions [32]. We also observed obvious apoptosis when NPCs were treated with IL-1β without any FBS. However, majority of NPCs went from adherent to floating when Ad-HO-1 transfected and subsequent experiments could not be carried out. When 1% FBS added, IL-1β can still induce apoptosis effectively, furthermore, Ad-HO-1 transfection and follow up experiments can proceed smoothly. Therefore, we used the treatment of IL-1β under 1% FBS condition to create the apoptosis model of NPCs. Though the specific reasons were not studied in this study, we considered that growth factors in FBS are beneficial to the maintenance of cell state.

HO-1 is the rate-limiting enzyme in heme catabolism with antioxidant, anti-inflammatory and anti-apoptotic activities [34]. HO-1 suppresses high glucose-induced apoptosis in podocytes by enhancing autophagy [35]. In this study, we demonstrate that levels of HO-1 and LC3-II/I levels are decreased in the IDD patients tissues compared to those from the LVF individuals. This suggests that apoptosis of the NPCs in the degenerative intervertebral disc tissues may be linked to decreased levels of autophagy. Moreover, HO-1 may be a key regulator of autophagy in the NPCs. Next, our results showed that HO-1 upregulation suppresses IL-1β induced apoptosis of NPCs by enhancing autophagy as shown by decreased P62 and increased LC3-II/I levels. Furthermore, immunofluorescence staining and TEM results show increased number of autophagosomes in NPCs transfected with Ad-HO-1 compared with controls. Moreover, HO-1 overexpressing NPCs treated with the 3-MA show increased P62 levels and apoptosis, and decreased expression of LC3II/I. These results indicate that HO-1-induced autophagy inhibits apoptosis in human NPCs.

Autophagy is regulated by mTOR-dependent and mTOR-independent pathways. Inhibition of mTOR activates autophagy in several cell types, including the NPCs [36, 37]. On the other hand, mTOR-independent pathways regulate autophagy in several cell types under specific conditions [26, 38]. We observed no significant changes in the phosphorylation status of mTOR during HO-1 induced autophagy. This suggests that HO-1 induced autophagy is not dependent on the mTOR pathway in the NPCs.

Class III Phosphoinositide 3-Kinase (PI3KC3) is one of the many PI3Ks that is required for autophagy initiation [23]. We demonstrate that HO-1 increases the formation of the Beclin-1/PI3KC3 complex. This suggests that HO-1 induces autophagy in a PI3KC3-dependent manner in the NPCs.

We also investigated the interactions between HO-1, autophagy and NF-κB by treating control and HO-1 overexpressing NPCs with 3-MA and CQ, which block PI3K and terminal process of autophagy, respectively. Our results revealed that HO-1 induced autophagy inhibits NF-κB in the NPCs. In a previous study, we showed that HO-1 reduced apoptosis of NPCs by inhibiting NF-κB [19]. Therefore, we confirmed that HO-1-induced autophagy in the human NPCs suppresses IL-1β-induced apoptosis by inhibiting NF-κB.

## MATERIALS AND METHODS

### Nucleus pulposus samples

Nucleus pulposus (NP) was obtained from 16 patients (6 females and 10 males) that underwent surgery for lumbar disc herniation and IDD-related low back pain; intervertebral disc (IVD) tissue samples were obtained from 6 patients (3 women and 3 men) with lumbar vertebral fractures (LVF) without any history of low back pain. Grading was done according to the Pfirrmann classification system using pre-operative MRI scans. The IDD patient samples were grades IV-V and the LVF patient samples were grade I-II.

We obtained written informed consent from all the tissue donors prior to surgery, and the study protocol was approved by the Ethics Committee of Chongqing Medical University (Chongqing, China).

### IHC staining

NP samples were fixed with 4% paraformaldehyde for 24 h, then embedded in paraffin, and cut into 4 mm thick sections. The IHC staining procedure was performed using the Streptavidin-peroxidase Immunohistochemical kit (Boster, Wuhan, China) according to the manufacturer's protocol. Briefly, the sections were treated with 3% H_2_O_2_ for 15 min at room temperature to eliminate endogenous peroxidase activity. Subsequently, the samples were incubated with 0.125% trypsin for 30 min at 37°C for antigen retrieval, and then blocked with normal goat serum for 15 min at room temperature. The sections were then incubated with the rabbit anti-HO-1 (Boster, Wuhan, China) and rabbit anti-collagen (Abcam, Cambridge, MA, USA) antibodies overnight at 4°C. Then, the sections were incubated with the goat anti-rabbit IgG-HRP (1:5000) antibody followed by counterstaining with hematoxylin.

### Primary NPC isolation and culture

Nucleus pulposus (NP) was harvested from the IVD tissues based on their morphology as visualized under a light microscope. Then, the NP samples were washed with PBS and incubated with 0.25% trypsin solution with 0.2% type II collagenase (Sigma, St. Louis, MO, USA) at 37 °C for 4~6 h. The tissue debris was removed by filtering through a 200-μm filter and the purified nucleus pulposus cells (NPCs) were cultured in DMEM/F-12 medium (HyClone, South Logan, UT, USA) supplemented with 10% FBS (Gibco, CA, USA), 100 μg/ml streptomycin, and 100 μg/ml penicillin at 37°C and 5% CO_2_ as described previously [32]. NPCs that were passaged twice were used for further *in vitro* experiments.

### Cell transfections

The recombinant human adenovirus vector overexpressing HO-1 (Ad-HO-1) and the control adenovirus vector (Ad-GFP) were obtained from Genecopoeia (Guangzhou, China. The negative control small interfering RNA (NC-siRNA) and siRNA targeting HO-1 (HO-1-siRNA) was purchased from Ambion (Foster City, CA, USA). For transfections, NPCs were seeded into 6-well plates, incubated for 24 h, and then transfected according to the manufacturer’s instructions. After subsequent treatments, the cells were harvested for analysis by flow cytometry, immunofluorescence, and western blotting.

### Western blot analysis

Human NP cells isolated from 16 IDD patients and 6 LVF patients were lysed on ice using RIPA Lysis Buffer (Beyotime, Wuhan, China). The total protein concentrations were determined using the Enhanced BCA Protein assay kit (Beyotime, Wuhan, China). Fifty micrograms of the total protein lysates were electrophoresed using 6–12% gradient SDS-PAGE gels. The separated proteins were transferred onto PVDF membranes and blocked with 5% nonfat dry milk in Tris-buffered saline (TBST) for 1 h. Then, the membranes were incubated with primary antibodies, including rabbit anti-HO-1 (Boster, Wuhan, China), rabbit anti-p-P65 (Phospho-Ser536; SAB, Maryland, USA), rabbit anti-Cleaved caspase 3 (SAB, CP, Maryland, USA), rabbit anti-Bax (SAB, Maryland, USA), rabbit anti-Bcl-2 (SAB, CP, Maryland, USA), rabbit anti-LC3B (CST, Boston, MA, USA), rabbit anti-P62 (Abcam, Cambridge, MA, USA), rabbit anti-Beclin-1 (Abcam, Cambridge, MA, USA), rabbit anti-PI3KC3 (Abcam, Cambridge, MA, USA), rabbit anti-Atg14 (CST, Boston, MA, USA), and rabbit anti-β-actin (Beyotime; Wuhan, China) overnight at 4°C The membranes were washed three times with TBST for 15 min and incubated with the anti-rabbit secondary antibody (Beyotime, Wuhan, China) at 37°C for 1 h. The membranes were developed and visualized using the ECL Plus Reagent (Beyotime, Wuhan, China). The results were analyzed using the SPSS 17.0 statistical software (IBM, Armonk, N.Y, USA).

### Flow cytometry

The cells undergoing apoptosis were analyzed using the Annexin V/PI apoptosis detection kit (LIANKE, Hangzhou, China). Briefly, 1 × 10^5^ degenerative human NPCs were seeded into each well of the 6-well culture plates. After the experimental treatments, the cells were harvested, washed twice with PBS, resuspended in the binding buffer (100 μl/1×10^5^ cells), and incubated with 5 μl of Annexin V-FITC for 20 min and 3 μl of PI (Hanbio, Shanghai, China) in the dark at room temperature for 15 min. Finally, 400ul binding buffer was added to cellular samples. The cells were analyzed by flow cytometry immediately after the staining was completed. The apoptotic rate was determined as the sum of the percentage of early (Annexin V^+^/PI^-^) and late apoptotic cells (Annexin V^+^/PI^+^).

### Immunofluorescence staining

The cells were fixed with 4% paraformaldehyde for 10 min and then treated with 5% Triton for 5 min. Then, the cells were stained with 1 μg/ml rabbit anti-SQSTM1/P62 (ab109012, Abcam, Cambridge, MA, USA) autophagosome marker antibody at 4°C overnight. Next, the cells were incubated with the anti-rabbit fluorescent secondary antibody (Proteintech, USA) at 37°C for 1.5 h. The cells were then counterstained with 4',6-diamidino-2-phenylindole (DAPI) and imaged using a fluorescence microscope (Leica, Germany).

### Transmission electron microscopy (TEM)

The NPC cells transfected with Ad-HO-1 or Ad-GFP were digested with trypsin and then centrifuged at 1200 rpm in 1.5 ml apical eppendorpf tubes. Then, after removing the supernatant, 2.5% glutaraldehyde was gently added along the side of the tube wall followed by conventional sample preparation process. Finally, 60 nm ultrathin sections were cut and analyzed using the Hitachi-7500 transmission electron microscope (Hitachi, Japan).

### Immunoprecipitation

For immunoprecipitation (IP), the cells grown in 10 cm cell culture dishes were harvested and incubated in the precooled IP lysis buffer for 30 min at 4°C. The resulting mixture was centrifuged at 14000g for 15 minutes. The supernatant was collected and the protein concentration of the samples was estimated using the Bradford method. Equal amounts of protein samples were incubated with rabbit anti-Beclin-1 (Abcam, Cambridge, MA, USA) primary antibody in a 1:100 dilution at 4°C and mixed constantly by inversion for 2 h. Then, 5 μl of protein A/G magnetic beads (Bimake, Houston, TX, USA) were added to the lysate and incubated overnight in an inverted position at 4°C in a magnetic rack (Bimake, Houston, TX, USA). Then, the lysate with the magnetic beads were centrifuged. The supernatant was removed. Then, 40-60 μl of the loading sample buffer solution was added to the magnetic beads and boiled for 10 min. The liquid supernatant was stored at−80°C for electrophoresis.

### Statistical analysis

All experiments were performed at least three times. The results are presented as the mean ± standard deviation (SD). Statistical analyses were performed using the SPSS 17 statistical software (SPSS Inc., IL, USA). The differences between the experimental groups were analyzed using the one-way analysis of variance (ANOVA) followed by Tukey’s test for comparisons between two groups; p<0.05 was considered statistically significant.
